# Manifestations of paediatric *Leishmania infantum* infections in Malta

**DOI:** 10.1016/j.tmaid.2010.11.005

**Published:** 2011-01

**Authors:** David Pace, Thomas N. Williams, Alicja Grochowska, Alexandra Betts, Simon Attard-Montalto, Michael J. Boffa, Cecil Vella

**Affiliations:** a Paediatric Infectious Diseases Clinic, Mater Dei Hospital, Msida, Malta; b Department of Paediatrics, Oxford and KEMRI Wellcome Trust Unit, Kilifi, Kenya; c Department of Haematology, Mater Dei Hospital, Msida, Malta; d Department of Histopathology, Mater Dei Hospital, Msida, Malta; e Department of Paediatrics, Mater Dei Hospital, Msida, Malta; f Department of Dermatology, Sir Paul Boffa Hospital, Floriana, Malta

**Keywords:** Paediatric leishmaniasis, *Leishmania infantum*, Phlebotomine sand fly, Malta

## Abstract

*Leishmania infantum* is endemic in the Maltese archipelago, a group of islands in the Mediterranean which are visited frequently by tourists from Northern European countries. The burden of leishmaniasis is highest in children who may present with cutaneous or visceral manifestations. We describe systematically the manifestations, diagnosis and management of leishmaniasis in children <14 years of age, who had a histopathological diagnosis of leishmaniasis in Malta, from 2004 to 2008. Eleven children were diagnosed with leishmaniasis; 8 children (15–44 months of age) had visceral disease and three (aged 9–13 years) suffered cutaneous infections. Prolonged high grade fever, pallor, hepatosplenomegaly, and pancytopenia were common presenting features of visceralisation. Diagnosis was based on the visualisation of amastigotes from bone marrow aspirates. Pentavalent antimonials were associated with treatment failure in two children, whilst liposomal amphotericin B was curative in all. Children with cutaneous leishmaniasis had dry crusted ulcero-nodular lesions on exposed areas which responded to intra-lesional instillation of sodium stibogluconate or to cryotherapy. Leishmaniasis should be included in the differential diagnosis of fever and hepatosplenomegaly or chronic cutaneous lesions in children who travel to Malta.

## Introduction

The leishmaniases are a group of vector-borne protozoan diseases caused by pathogenic *Leishmania* species which, if symptomatic, result in protean clinical manifestations that range from localised cutaneous ulcers to disseminated lethal infection. Such diversity is complicated by anthroponozoonotic modes of transmission which follow a sylvatic, peridomestic or anthroponotic cycle depending on the mammalian reservoir.^1^ Being widespread in all continents (except Australia and Antarctica), but primarily concentrated in South Asia, the Horn of Africa, Central and South America and endemic in the Mediterranean basin, these parasitoses are a major public health concern.^2^ Global annual incidence is estimated at 1.5 million cases of cutaneous disease and 500,000 cases of visceral leishmaniasis (VL), with prevalence rates reaching 12 million.^2^ Leishmaniasis is estimated to result in a loss of 1.97 million disability adjusted life years (DALYs) worldwide, classifying third from all vector-borne infections.^3^ In Southern Europe annual incidence rates of VL, ranged from 0.11 to 8.32/100,000 population from 1998 to 2007.^4^ In non-endemic countries leishmaniasis is a disease of travellers and migrants.^5^

Leishmaniasis is endemic in the Maltese archipelago, a group of small islands (consisting of Malta, Gozo and Comino) in Southern Europe with an area of 315 km^2^, a population of around 400,000 inhabitants and a population density of 1285 residents per km^2^, the highest in the European Union.^6^ Malta is visited by an average of 1.2 million tourists per year, most of whom come from northern European countries, namely UK and Germany.^6^ Cutaneous and visceral leishmaniases in Malta are caused by a single species, *Leishmania infantum*, which is transmitted from dogs to humans by one local species of sand fly, *Phlebotomus perniciosus*.^7^ The few studies performed in Malta have not identified any other *Leishmania* species in humans who acquired cutaneous disease in Malta, or in sand flies, dogs or rats^8,9^ and because of limited resources species identification from clinical specimens is not carried out as yet.

In Malta two zymodemes (strains with different isoenzyme profiles) of *L. infantum* have been characterised: MON1 that causes VL and, as in other Mediterranean countries, is the commoner form, and MON78 which is dermotropic,^8^ but which may cause VL in the immunocompromised.^10^ Notification of leishmaniasis has been compulsory in Malta since 1946, however LCL had not been recognised prior 1981 and no cases were notified before 1983.^11^ By contrast, VL had been recognised since 1909,^12^ with the largest number of cases (207), being recorded in 1948.^13^ The quasi-eradication of stray dogs, improved sanitary conditions and urbanisation have resulted in a drastic decrease in the incidence of VL following the Second World War, from 67/100,000 population in 1948 to 8.2/100,000 population in 1955 and down to a mean of 1.1/100,000 over the last 10 years.^13^ Rates of cutaneous disease have remained low since 1983 (mean 1.87/100,000),^14^ however, being a non-life-threatening illness and often not notified or unrecognised, the incidence of LCL is confounded by underreporting. Children <3 years of age suffer the majority of the visceral disease burden.^15^ The case fatality rate is extremely low with only two individuals, aged >65 years, dying from VL since 1991.^16^ Leishmaniasis has not yet been eradicated due to persistent transmission from canine leishmaniosis,^9^ the only identified reservoir in Malta, with 31% (406/1310) of all indirect immunofluorescence (IF) tests carried out from 2005 to 2008 on dogs with suspected leishmaniosis being positive.^17^ The prickly pear (*Opuntia vulgaris*) and rubble walls, which are widespread in Malta, create a perfect habitat for the breeding of sand flies.^18^

The female phlebotomous sand fly, a 2–3 mm arthropod belonging to the genus *Phlebotomus* in the Old World is a noiseless flier which feeds from dusk throughout the night.^19^ Outdoor activities are common in Malta during hot summer nights (the peak tourist season), putting unaware individuals at risk of being bitten by *P. perniciosus*, whose probing activity is increased when infected.^20^ Age related differences in T-cell immunity might contribute to the high disease burden in children <3 years old. Seasonal variation of leishmaniasis has not been observed in Malta due to its variable incubation period (6 weeks–6 months but at times as long as 10 years).^15^

We describe the manifestations, diagnosis and management of leishmaniasis in children, diagnosed in Malta over a 5 year period, from 2004 to 2008, in order to make physicians in non-endemic countries aware of the persistence of leishmaniasis in this Mediterranean country. Leishmaniasis has a wide differential diagnosis and is an important treatable parasitosis that should be considered in paediatric travellers returning from endemic regions.

## Methods

The hospital records of all children (<14 years) with a histopathological diagnosis of leishmaniasis from 2004 to 2008, who were identified from the database of the Pathology Department at Mater Dei Hospital which is the main acute general hospital in Malta, were reviewed. Eight children (aged 15–44 months) presented with visceral leishmaniasis, of which 7 were female. Six children were Maltese (4 from Malta and 2 from Gozo), and 2 were Somali (Table 1). The two refugees from Somalia were a boy aged 44 months, who had been living in Malta for the previous 11 months, and an 18 month old girl who had been in Malta for 1 year. Three Maltese children (aged 9–13 years) had a definite histological diagnosis of cutaneous leishmaniasis (Table 2). All were male and one lived in Gozo. A single ulcerated nodule was noted below the left lower eyelid in the 13 year old boy (Case 10) and on the right arm in the 11 year old boy (Case 11). The 9 year old Gozitan boy (Case 9) had multiple lesions on his face and right lower limb.

## Results

### Visceral leishmaniasis

#### Clinical features

Prolonged high grade fever was observed consistently in all 8 children presenting with VL. Other symptoms included anorexia, irritability, cough, rhinorrhoea, vomiting and diarrhoea. All appeared pale and hepatosplenomegaly was detected in all, except in Case 5, who only had splenomegaly. None exhibited prominent lymphadenopathy. Most children appeared well between episodes of fever and none had signs of septic shock or disseminated intravascular coagulation.

#### Laboratory findings

**Figure 1:**
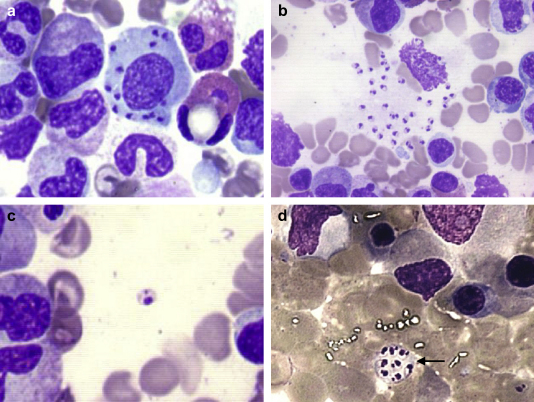
Bone marrow smears from Case 3 (Giemsa stain ×1000). a) Intra-macrophageal Leishman-Donovan bodies; b) Ruptured macrophage with release of several amastigotes, c) Extracellular amastigote with prominent nucleus and kinetoplast, d) Cyst-like structure (arrow) containing amastigotes.

Source: Haematology Department, Mater Dei Hospital, Malta.

Pancytopenia was a frequent but inconsistent finding with total white cell counts of 2.3–5.9 × 10^9^/l, neutrophils as low as 0.22 × 10^9^/l (range 0.22–2.8 × 10^9^/l), haemoglobin concentration of 5.3–9.5 g/dl with microcytic, hypochromic red cells, and platelet counts of 34–157 × 10^9^/l. High C-reactive protein (range: 29–207 mg/l), an ESR >40 mm/h, hypoalbuminaemia and negative blood cultures were observed consistently. A bone marrow aspirate was diagnostic in all (Fig. 1) and excluded any underlying haematological malignancy. Serological testing using an IF assay for anti-*L. infantum* IgG (*Leishmania-Spot IF*, bioMérieux, Marcy l’Etoile, France)^21^ and/or an indirect enzyme-linked immunosorbent assay (ELISA) for anti-*L. infantum* IgG + IgM (Vircell, S.L., Granada, Spain)^22^ were performed. All children tested had a positive IF assay but ELISA was falsely negative in Case 7 who, subsequently, was confirmed to be infected with *L. infantum* using a polymerase chain reaction (PCR) assay detecting the species specific genome regions SSUrRNA and ITS-1. The two Somali refugees were presumed to have acquired the infection in Malta; however, species identification was not performed. Anti-nuclear antibodies were detected transiently in case 3.

#### Treatment

Sodium stibogluconate (*Pentostam*, GlaxoSmithKline, Middlesex, UK) at a dose of 20 mg/kg for 21 days (preceded by an initial test dose of 25 mg) in combination with oral allopurinol (20 mg/kg daily for 10 days) was the treatment of choice prior 2007. Liposomal amphotericin B (L-AmB, *AmBisome*, Gilead Sciences Int. Ltd, Cambridge, UK) at a total dose of 20–30 mg/kg administered over 10 days, was preferred subsequently in view of concerns on treatment failure with antimonials. Most children received a blood transfusion for anaemia since the haemoglobin concentration is known to drop in the first 2 weeks of treatment.^15^ The microcytosis, possibly resulting from associated iron deficiency, generally resolved after treatment and none had an underlying haemoglobinopathy. Case 1 needed multiple platelet transfusions due to recurrent epistaxis as a result of thrombocytopenia (dropping to 2 × 10^9^/l) associated with treatment failure. Because of the underlying neutropenia, many children were started on antibiotics on admission. The caMRSA bacteraemia in Case 2, resulting from an infected intravenous cannula, was associated with an appropriate neutrophilic response.

#### Response to treatment

Successful treatment resulted in defervescence (noted within 2–8 days) which coincided with a rise in the platelet count, whilst unremitting fever or its recrudescence, a decreasing platelet count or non-resolving hepatosplenomegaly indicated treatment failure (Case 1: a 15 month old Maltese girl and Case 2: a 27 month old girl from Gozo: Table 1). Hospital stay for children showing an immediate response was short (4–11 days) and the full treatment course was subsequently completed as outpatients. Because of the language barrier and social background, the two Somali children were kept in hospital to ensure treatment compliance. Treatment failure was associated with a prolonged hospital stay (18–30 days). Despite their documented toxicities, no complications were associated with antimonials and the hypokalaemia observed in Case 1 was induced by L-AmB. All children were followed up for 6–12 months and none developed any clinical signs of relapse.

### Localised cutaneous leishmaniasis (LCL)

#### Clinical presentation

**Figure 2:**
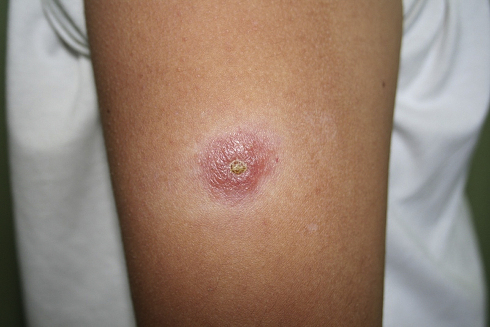
Cutaneous leishmaniasis (Case 11): Raised erythematous nodule (diameter of 1 cm) with central dry ulceration and surrounding inflammatory hypopigmentation on the right lateral arm.

Source: MJB, Dermatology Department, Sir Paul Boffa Hospital, Floriana, Malta.

The three boys presenting with cutaneous disease had crusted ulcero-nodules (<1.5 cm in diameter) on exposed areas namely on the face, arm (Fig. 2) and legs, with Case 9 having multiple lesions (Table 2). Diagnosis was delayed in Cases 9 and 10 for 3 months and 2 years, respectively since the disease was not recognised, whilst Case 11 was promptly diagnosed at presentation. Case 9 received local antimicrobial treatment for suspected fungal lesions and was also prescribed oral flucloxacillin for possible impetigo. Similar treatment was administered to Case 10 on whom incision and curettage of the ‘cyst-like’ lesion was also inappropriately attempted with no success.

#### Laboratory findings

**Figure 3:**
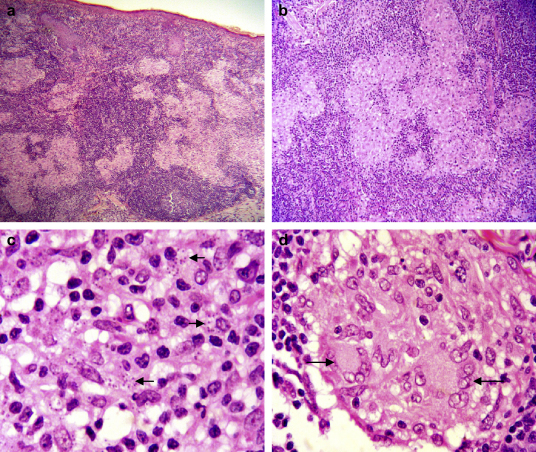
Histology of cutaneous leishmaniasis in Case 10 (H&E stain): a) Diffuse chronic inflammatory cell infiltrate in dermis with multiple non-caseating granulomata (×40); b) Tuberculoid-type granulomata with central histiocytes and peripheral inflammatory cells (×100); c) Leishman-Donovan bodies (arrows) within cytoplasm of epithelioid histiocytes (×600); d) Langhans type giant cells (arrows) within a granuloma (×600).

Source: Pathology Department, Mater Dei Hospital, Malta.

Histological examination of tissue specimens was diagnostic in all. Diagnosis of LCL in Case 10 (Fig. 3) was only clinched after his skin lesion was ultimately excised, 2 years after onset, for psychological distress. Slit skin smears performed on case 11 did not reveal the *Leishmania* amastigotes, however, similar to Case 9, a punch biopsy of the lesion was diagnostic.

#### Treatment

The management of all three cases was different: Case 9 received cryotherapy with liquid nitrogen in view of the multiplicity of his lesions, the surgical procedure carried out on Case 10 was curative and Case 11 received repeated intra-lesional injections of sodium stibogluconate. All treatment modalities resulted in clinical cure and although Case 10 had a residual surgical scar he had no recurrence.

## Discussion

The clinical manifestations of the children described in our study are typical of the presentation of childhood VL in developed countries in the Mediterranean littoral.^23–26^ By contrast, VL in children in Albania, a less industrialised Mediterranean country with a much higher incidence rates of VL (reaching up to 25/100,000 in 0–6 year old children), is more frequently complicated by concurrent infections such as bronchopneumonia and diarrhoea.^27^ This variable clinical expression of leishmaniasis not only depends on the inoculated zymodeme and efficacy of the immune response, but is also affected by environmental factors and the genetic constitution of the host.^28^ Cryptic infections caused by viscerotropic *Leishmania* species are also common.^29^

The hepatosplenomegaly observed in our children with VL is a result of the accumulation of mononuclear phagocytic cells which causes hyperplasia of reticulendothelial cells. Pancytopenia occurs secondary to bone marrow involvement and hypersplenism. Concurrent viral infections and occasionally life-threatening bacterial sepsis may occur secondary to the associated immunosuppression.^30^ Untreated VL is fatal within 2–3 years. Persistence of *Leishmania* is characteristic, and although none of the children in our case series relapsed, relapses can potentially occur up to 6–12 months after treatment.

Diagnosis of VL in our study was clinched by demonstration of amastigotes by light microscopy of bone marrow smears, with all children having intracellular amastigotes, known as Leishman-Donovan bodies (Fig. 1). Interestingly, an extracellular cyst-like structure (Fig. 1d) was noted only in children receiving antibiotics at the time of sampling. The well defined circular outline of the structure, as well as the regular arrangement of the amastigotes within and the staining characteristics of the interspersed material, makes it unlikely to represent cytoplasmic fragmentation of a macrophage that may occur during smearing. This finding has never been described previously and although its origin is unexplained, could plausibly be a response to an adverse milieu created by the antibiotics. Electron microscopy could perhaps elucidate its morphology and significance.

Although not performed in our study, light microscopy of splenic or lymph node smears may alternatively be used for diagnosing VL. We avoided splenic aspirates due to the potential risk of death from massive bleeding associated with thrombocytopenia, a frequent haematological manifestation in Maltese children with VL. Lymph node smears were not indicated as none of the described children had lymphadenopathy. *Leishmania* may be cultured on a Novy-McNeal Nicolle medium but this is not done routinely in several countries due to the required expertise and cost. Despite lacking standardisation conserved sequences in minicircle kinetoplast DNA or in the small subunit rRNA gene of *Leishmania* may be detected rapidly by PCR on lymph node and bone marrow aspirates (Case 7), on peripheral blood^31^ or on urine.^32^

Some of our children with VL had anti-*Leishmania* antibodies which are useful diagnostically but are not protective, probably due to the obligate intracellular nature of the parasite. Their detection by IF, ELISA or Western blot must be correlated with clinical findings since false positives may occur in asymptomatic or resolved *Leishmania* infections and in other infectious diseases.^33^ Molecular mimicry of *Leishmania* antigens, in addition to polyclonal B-cell activation, may result in the production of autoantibodies to ribonucleoproteins (Case 3), rheumatoid factor and smooth muscle, and may be responsible for a positive Coombs’ test (Case 6).^34,35^ Autoantibodies, which are generally non-pathogenic, may play a yet undefined role in protection but can cause diagnostic confusion with connective tissue disorders.^36^ The detection of serum anti-rK39 (an amino acid repeat that is conserved within the *L. donovani* complex) IgG^37^ or low molecular weight antigen (LMWA), thought to be a carbohydrate antigen derived from amastigotes, in urine^38^ are alternative tests used for the rapid diagnosis of VL.

**Figure 4:**
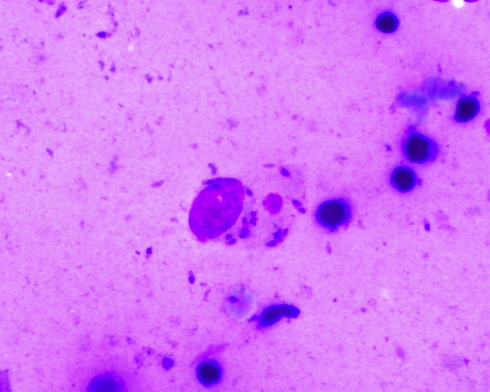
Cytology of slit skin smear showing extracellular and intracellular (within epithelioid histiocyte) amastigotes (Giemsa stain ×600).

Source: Pathology Department, Mater Dei Hospital, Malta.

The Maltese children with LCL had ‘dry’ nodulo-ulcerative lesions (Fig. 2) typical of *L. donovani* complex disease. Diagnosis was based on the visualisation of amastigotes on direct microscopy of skin smears (Fig. 4) or of punch/ellipse skin sections (Cases 9–11). Because of its benign presentation, LCL in children in Malta tends to be treated in the community with cryotherapy without an attempt for a histopathological diagnosis and without notification. PCR has improved the sensitivity of microscopy and can identify the infecting species, but is costly and not done in Malta.^39^ The utility of serology is limited due to the low titres of antibodies induced in LCL and therefore we do not perform anti-*Leishmania* antibodies for children with LCL.^39^ The leishmanin (Montenegro) skin-test, reflecting cell-mediated immunity, may be used but cannot distinguish between past and present infection.^39^

Parenteral pentavalent antimonials have been the standard treatment for both cutaneous and visceral disease for the last 50 years; however, the associated toxicity, long treatment duration (20–28 days) and the development of resistance from irregular compliance have led to the utilisation of alternative drugs.^40^ As in Malta, lipid formulations of amphotericin B are nowadays more commonly used in Southern Europe due to their cost effectiveness when administered in short course regimens that results in a reduced hospital stay which offsets their high cost.^41^ Alternative agents such as the aminoglycoside paromomycin, is expected to provide a cheaper but efficacious alternative to amphotericin B.^42^ Furthermore, miltefosine, the only oral formulation that has proven efficacy in children,^43^ may be used as outpatient therapy to treat VL, although non-compliance is a concern.

Although cutaneous lesions may be observed expectantly as most would heal spontaneously within months, time to resolution varies between species and individuals. Intra-lesional antimony regimens administered between 1 and 3 times weekly for 2–8 weeks (Case 11) are effective, but painful. The local application of paromomycin ointment,^44^ cryotherapy^45^ or thermotherapy,^46^ are attractive options devoid of systemic side effects, however, the success rate is variable and species dependent. Prolonged and cosmetically significant or multiple lesions may be treated with parenteral antimonial compounds, amphotericin B, petamidine and oral miltefosine. Most of these treatment options have been poorly investigated in clinical trials and the need for proper research assessing the long term effects of such regimens, particularly in children, is still there.^47^

Being an island there are several public health measures that can be implemented effectively to eradicate leishmaniasis from Malta. Control measures on the importation of dogs are already in place making it very unlikely for the introduction of *L. infantum* from neighbouring endemic Mediterranean countries. However, the current veterinary practice of treating infected pet dogs with antimonials severely limits the control of canine leishmaniosis, since dogs are known to have high rates of relapse.^48^ In addition such practice promotes drug resistance^48^ and is very likely the reason for the treatment failures with antimonials seen in some of the children in our study. In the absence of a culling program for infected dogs prospects for eradication of leishmaniasis are bleak, although its implementation is likely to be met with resistance from dog owners and animal rights groups.

Conversely, the promotion of deltamethrin-impregnated dog collars would be effective in preventing canine leishmaniosis and may result in a reduction in VL.^49^ Although vaccine prevention of leishmaniosis in dogs by means of *Leishmune*^®^, the only licensed canine vaccine that is currently in use in Brazil is attractive, its impact on the epidemiology of zoonotic VL in humans is still unknown.^50^

Vector control by indoor residual spraying of insecticides is unpopular^51^ and only has a transient effect due to the predominant exophagic and exophilic nature of most phlebotomous sand flies, whilst outdoor spraying is ineffective.^19^ Breeding ground destruction by removal of rubble walls is illegal since these are part of the Maltese heritage and are protected. In Malta the wide distribution of *Opuntia* trees found growing wildly or else being cultivated for their fruit from which a characteristic liqueur is also manufactured, makes their eradication challenging.

At present avoiding sand fly bites is the most practical advice that can be given to tourists visiting Malta in order to prevent leishmaniasis. The application of insect repellents on children who will be spending time outdoors after dusk and before dawn, as is common during recreational activities in summer in Malta, is encouraged. For those who prefer to sleep with the windows open, insect screens are recommended as these will help prevent getting bitten by sand flies indoors during sleep. Sleeping in storeys above the first floor is effective in avoiding sand fly biting as sand flies are poor fliers and can only hop a vertical distance of 1 m.^52^ Immunisation against leishmaniasis is not possible at present since despite a century of research no effective vaccine is available to protect against human leishmaniasis.

## Conclusion

The manifestations of leishmaniasis described in this case series highlight the persistence of this parasitosis in Malta. The small contained size of Malta creates an opportunity for eradication of leishmania which is, however, hindered by the persistence of chronically infected dogs. Clinical, epidemiological and entomological studies of leishmaniasis in this country are needed. Eliminating the threat of leishmaniasis would not only be beneficial for the Maltese population but also for the tourist industry which is a major source of income for Malta. Unfortunately leishmaniasis is perceived as non-profitable by pharmaceutical companies and failing industrial interest and investment in research, especially in developing countries, leishmaniasis will remain a neglected disease.

## Author’s contribution statement

DP and TNW designed the study. DP, AG, AB, SAM, MJB and CV were involved in the acquisition, analysis and interpretation of the data. DP drafted the article which was critically revised for important intellectual content by TNW, AG, AB, SAM, MJB and CV.

## Conflict of interest

None declared.

## Figures and Tables

**Table 1:** Presentation and management of children with visceral leishmaniasis (2004–2008).

Case number	Year	Age/months	Sex	Presentation	Investigations	Treatment	Blood products	Complications	Hospital stay/days
Case 1 Maltese	2004	15	F	Febrile up to 40.5 °C for 10 days Pallor Hepatosplenomegaly	Pancytopenia CRP: 143 mg/l BM: LDBs	Sodium stibogluconate + allopurinol × 10 days (switched to *Ambisome* [L-AmB] after 20 days)	PC Platelets albumin	• Declining platelet counts down to 2 × 10^9^/l, causing epistaxis, diminishing haemoglobin concentration despite transfusion, non-resolving hepatosplenomegaly, development of ascites and persistence of fever >38 °C after 20 days of antimonials indicated treatment failure: switched to L-AmB for 10 days (20 mg/kg) • L-AmB induced hypokalaemia	30
Case 2 Gozitan	2005	27	F	Febrile up to 39.4 °C for 7 days Pallor Hepatosplenomegaly Pharyngitis	Anaemia Neutropenia CRP: 207 mg/l aELISA IgG/IgM: 20.31 BM: LDBs	Sodium stibogluconate + allopurinol × 10 days (switched to L-AmB after 14 days)	PC	• Generalised macular rash and recurrence of fever after 10 days of antimonials associated with pus oozing from cannula site. Diagnosed with caMRSA bacteraemia and treated with teicoplanin for 10 days • Non-resolving fever 5 days through teicoplanin and enlarging spleen indicated treatment failure: antimonials switched to L-AmB for 10 days (30 mg/kg)	18
Case 3 Somalia (in Malta for 11 months)	2006	44	M	Febrile up to 40 °C for 7 days Diarrhoea Pallor Hepatosplenomegaly	Anaemia Thrombocytopenia CRP: 132 mg/l IF IgG + ve, BM: LDBs	Sodium stibogluconate for 21 days Allopurinol for 10 days	PC	• ANA pos 1:80 (fine speckled nucleolar pattern): not detected 3 months later	24
Case 4 Gozitan	2007	28	F	7 day h/o fever up to 40°C Clear rhinorrhoea Miserable Pallor Hepatosplenomegaly	Anaemia Thrombocytopenia CRP:187 mg/l, ESR:72 mm/h IF IgG: +ve BM: LDBs	Sodium stibogluconate for 28 days Allopurinol for 10 days	Nil	Nil	11
Case 5 Maltese	2007	19	F	Febrile up to 39 °C for 4 days Pallor Splenomegaly	Pancytopenia: CRP:100 mg/l, ESR: 56 mm/h ELISA IgG/IgM: 20.3, BM: LDBs	Sodium stibogluconate for 21 days Allopurinol for 10 days	PC	Nil	5
Case 6 Maltese	2007	16	F	Recurrent URTIs during the previous 4 months Poor weight gain, Febrile up to 38.2 °C in hospital, Irritable Pallor Hepatosplenomegaly	Pancytopenia Direct Coombs test: +ve BM: LDBs	L-AmB for 10 days (30 mg/kg)	PC	Nil	4
Case 7 Maltese	2008	20	F	Febrile up to 40.2 °C for 7 days Rigors Irritable Pallor Hepatosplenomegaly	Pancytopenia CRP 29 mg/l IF IgG + ve ELISA IgG/IgM–ve BM: LDBs PCR: *L. infantum* +ve	L-Amb for 10 days (20 mg/kg)	Nil	Nil	11
Case 8 Somalia (in Malta for 1 year)	2008	18	F	Febrile up to 40.3 °C for 3 weeks Refusing to walk Pharyngitis, Hepatosplenomegaly	Pancytopenia CRP 114 mg/l IF IgG +ve ELISA IgM/IgG: 20.7 BM: LDBs	L-Amb for 10 days (20 mg/kg)	PC	Nil	11

*Abbreviations*: M: male, F: female, URTI: Upper respiratory tract infection, CRP: C-reactive protein, BM: Bone marrow aspirate, LDBs: Leishman-Donovan bodies, ELISA: Enzyme-Linked Immunosorbent Assay, IF: Indirect immunofluorescence assay for *L. infantum* IgG; ESR: Erythrocyte sedimentation Rate, PCR: polymerase chain reaction, PC: packed cells, ANA: Anti-Nuclear Antibodies.

a Values for ELISA represent an antibody index with a cut off >11 taken as positive.

**Table 2:** Presentation and management of children with cutaneous leishmaniasis (2004–2008).

Case number	Year	Age/years	Sex	Presentation	Investigations	Histology	Treatment
Case 9 Gozitan	2005	9	M	2 month h/o multiple enlarging round nodules on lateral border right eye, right temporal area, left cheek, left pinna, right thigh and knee. Lesions crusted in centre and occasionally oozed.	Punch biopsy form lesions on temple and cheek	Several granulomata and LDBs in histiocytes	Cryotherapy ×2, at a 2 month interval, using liquid nitrogen
Case 10 Maltese	2007	13	M	2 year h/o painless crusted ulcerated nodule 1.4 cm in diameter, below the left lower eyelid	Excised under local anaesthesia	Granulomatous inflammation with LDBs	No further treatment
Case 11 Maltese	2008	11	M	3 month h/o painless crusted ulcerated nodule, 1 cm in diameter, on the lateral aspect of the right arm	Slit skin smears followed by punch biopsy	Cytology of skin smears: lymphocytes, macrophages and multi-nucelated giant cells Biopsy: tuberculoid-type granulomas, scattered LDBs	intra-lesional sodium stibogluconate ×4 at 5 day intervals

*Abbreviations*: M: male, h/o: history of; LDBs: Leishman-Donovan bodies.
